# The critical role of γ-secretase and its inhibitors in cancer and cancer therapeutics

**DOI:** 10.7150/ijbs.87334

**Published:** 2023-10-02

**Authors:** Congkuan Song, Jinjin Zhang, Chenzhen Xu, Minglang Gao, Ning Li, Qing Geng

**Affiliations:** 1 Department of Thoracic Surgery, Renmin Hospital of Wuhan University, Wuhan, China.; 2 Department of Emergency, Taihe Hospital, Shiyan, China.

**Keywords:** γ-secretase, γ-secretase inhibitors (GSIs), cancer

## Abstract

As a multi-substrate transmembrane protease, γ-secretase exists widely in various cells. It controls multiple important cellular activities through substrate cleavage. γ-secretase inhibitors (GSIs) play a role in cancer inhibition by blocking Notch cleavage, and are considered as potential therapeutic strategies for cancer. Currently, GSIs have encouraging performance in preclinical models, yet this success does not translate well in clinical trials. In recent years, a number of breakthrough discoveries have shown us the promise of targeting γ-secretase for the treatment of cancer. Here, we integrate a large amount of data from γ-secretase and its inhibitors and cancer in nearly 30 years, comb and discuss the close connection between γ-secretase and cancer, as well as the potential and problems of current GSIs in cancer treatment. We analyze the possible reasons for the failure performance of current GSIs in clinical trials, and make recommendations for future research areas.

## Introduction

γ-secretase is a multi-substrate transmembrane protease associated with Alzheimer's disease (AD), and is widespread in a variety of cells. It consists of four different integral membrane proteins: presenilin (PS1 or PS2), anterior pharynx-defective 1 (APH1A or APH1B), presenilin enhancer protein 2 (Pen-2), and nicastrin, containing 20 transmembrane domains (TMDs) and a large extracellular domain (ECD). γ-secretase engages in various biological pathways through substrate cleavage. Inhibition of γ-secretase activity has been considered as a potential therapeutic strategy for cancer. γ-secretase inhibitors (GSIs) have shown encouraging performance in preclinical models; however, their performance in clinical trials has been unsatisfactory. This may be partly attributed to a poor understanding of the γ-secretase and its inhibitors.

In recent years, Yigong Shi et al. analyzed the structure of γ-secretase (including the sequence of each subunit) and the cryo-electron microscopic structure of γ-secretase binding Notch and amyloid precursor protein (APP) under the condition of high-resolution by using ultra-low temperature electron microscopy 1-4, and also reported for the first time the four atomic resolution cryo-electron microscopic structure of γ-secretase binding three small molecule inhibitors and one regulator, elucidating the molecular mechanism of γ-secretase in recognizing different kinds of inhibitors and modulators 5. These breakthrough findings are exciting and will greatly advance the design and optimization of the next generation of γ-secretase inhibitors and modulators. We seem to see promising prospects for targeting γ-secretase against various human diseases, including cancer. Therefore, it is necessary to further expand our understanding on the critical role of γ-secretase and its inhibitors in cancer and cancer therapeutics.

## The components and functions of γ-secretase complex

**Figure 1:**
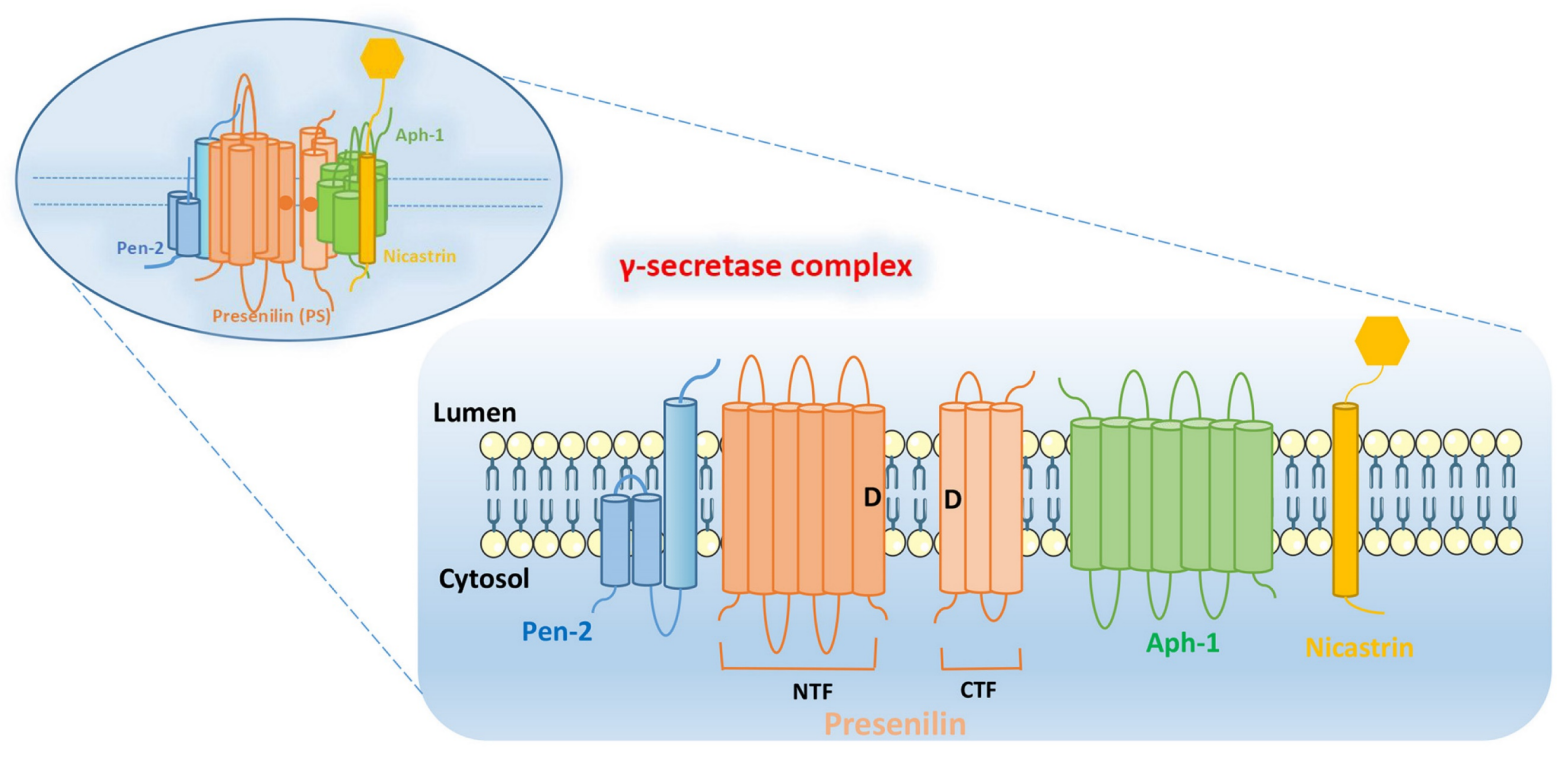
Structural integrity of γ-secretase complex. It is composed of four subunits: PS, Pen-2, Aph-1, and Nicastrin. PS can be decomposed by endogenous proteins between the 6th and 7th TMDs into two parts: N-terminal fragment (NTF) and C-terminal fragment (CTF). The aspartate residues (Asp257 and Asp385: as shown by the “D” in Figure) in the 6th and 7th TMDs are essential for their enzymatic activity.

γ-secretase complex is composed of four subunits: PS, Pen-2, Aph-1, and Nicastrin. The subunits of γ-secretase are closely arranged and each subunit contains at least one TMD. The intact γ-secretase also contains the glycation part of the ECD in Nicastrin subunit (**Figure 1**).

**PS:** PS is found in mammals in two subtypes PS1 and PS2, encoded by PSEN1 and PSEN2, respectively 6, 7. PS contains 9 TMDs, which are decomposed by endogenous proteins between the 6th and 7th TMDs into two parts: N-terminal fragment (NTF) and C-terminal fragment (CTF). The aspartate residues (Asp257 and Asp385) in the 6th and 7th TMDs are essential for their enzymatic activity. The precursor of PS1 is an inactive holoprotein, which is subsequently hydrolyzed to a heterodimer composed of PS1-CTF and PS1-NTF with the synergistic action of other subunits 8, 9. The whole-protein form of PS1 is barely detectable in the organism, while the catalytically active PS1 is often present as a heterodimer. Notably, not all of the catalytically active PS1 exists as a heterodimer, such as ΔE9 PSEN1^10^-^12^.

**Pen-2:** Pen-2, encoded by PSENEN, was previously thought to be a “U-shaped” hairpin protein with N- and C-terminus exposed to the lumen, containing two TMDs 13, 14. However, further researches have yielded different findings. These studies 15, 16 found that Pen-2 harbors three TMDs, two of which traverse the membrane only half-way from the intracellular side, with N-terminus of Pen-2 facing the cytoplasm and the C-terminus exposed to the lumen (**Figure 1**). Pen-2 is closely linked to PS, facilitating the automatic catalytic cleavage of PS (located between TMD6 and TMD7) to produce two fragments, NTF and CTF 10, 17, 18. The C-terminal hydrophilic region of Pen-2 was reported to be critical for stabilizing PS1-NTF and -CTF, but it is not necessary 16. And HP1 (the first of the two hydrophobic regions contained by Pen-2) is essential for determining the topology of Pen-2, which is required for promoting Pen-2-mediated endoproteolysis of PS1 and γ-secretase activity 19.

**Aph-1:** Aph-1 can be encoded by APH1A or APH1B, and contains seven TMDs, with the N-terminal domain facing the lumen and the C-terminal domain facing the cytoplasm. Together with Nicastrin, it plays a supporting role in facilitating the assembly and transport of γ-secretase complex. At the same time, it is also responsible for supporting the proteolytic activity of γ-secretase 20, 21.

**Nicastrin:** Nicastrin, a glycoylated protein encoded by NCSTN, contains a large ECD and is thought to serve as a complement of enzyme substrate, which can provide docking sites for γ-secretase substrates 22, 23. In addition, Nicastrin binds well to both NTF and CTF of PS, and is the component that maintains the stability of PS, and also have to rely on the PS to leave the endoplasmic reticulum (ER) to reach the cell surface 9, 24.

Overall, γ-secretase complex contains twenty TMDs and a large ECD from Nicastrin 4. PS is the active center of γ-secretase, and the other three subunits (Nicastrin, Aph-1 and Pen-2) are essential components for the maturation and stability of γ-secretase 3. These four subunits have different functions, and they need to be properly assembled, modified, matured and transported to the corresponding sites to play their normal physiological functions. They interact with each other to play hydrolytic activity and shear function together.

## The γ-secretase substrates associated with cancer

**Figure 2:**
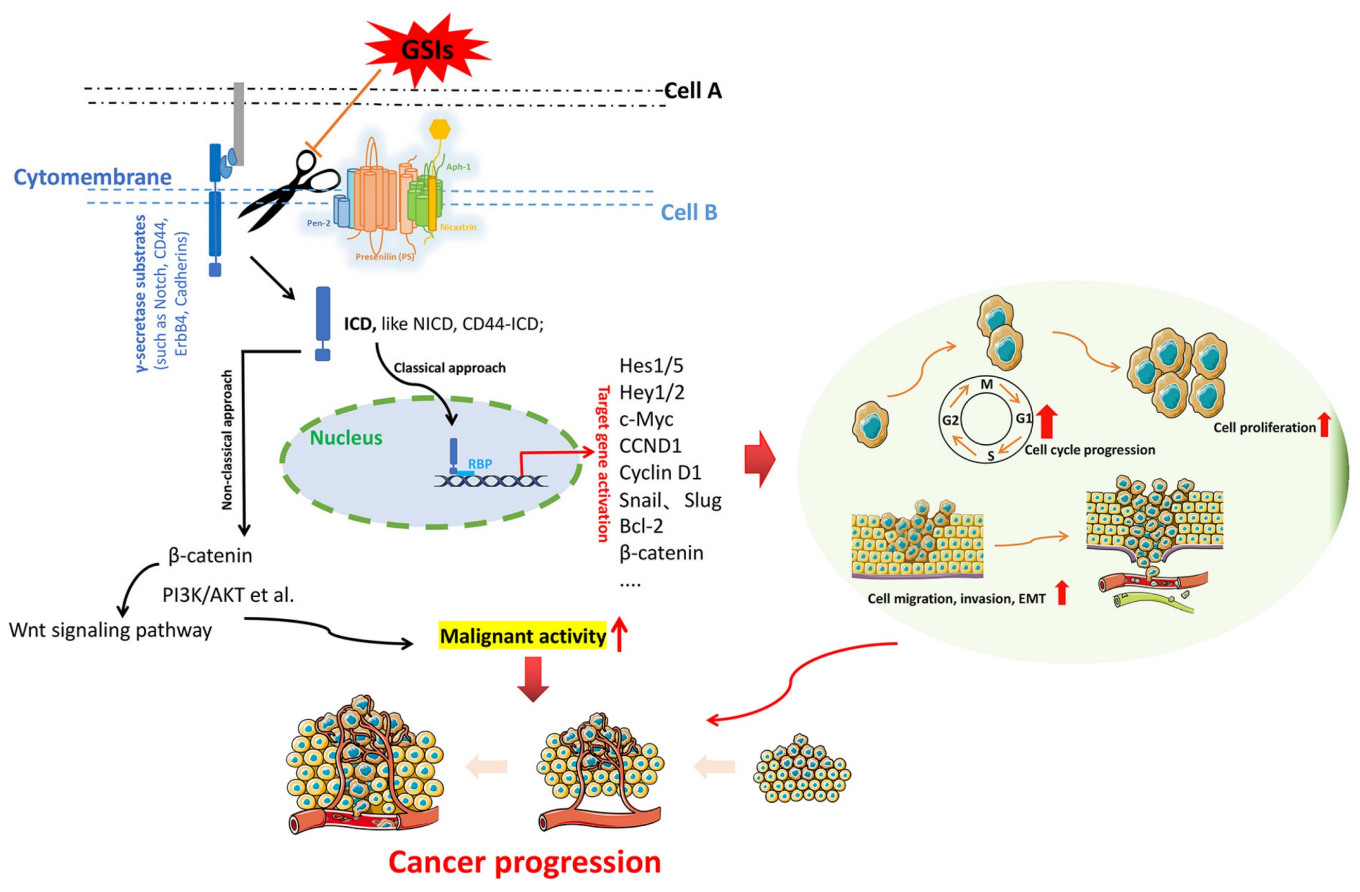
The fundamental process by which γ-secretase acts on substrates to exert essential functions. γ-secretases cleave the key substrate (e.g. Notch, CD44, ErbB 4, Cadherins) to release an active ICD, which allows ICD to migrate to the nucleus, where it binds to the transcription factors (e.g. CSL and MAML) to activate downstream effector factors. Activation of downstream target genes triggers multiple oncogenic pathways, which triggering cancer progression.

γ-secretase is a kind of multi-substrate protease complex widely existing in various cells. It is mainly involved in the cleavage and hydrolysis of a variety of transmembrane proteins, such as APP, Notch, ErbB4, CD44, Cadherins, etc 25. The intracellular domains (ICDs) of these substrates are released from the membrane into the cytoplasm under the action of γ-secretase, and these ICDs have different physiological functions associated with regulating the transcription of downstream genes. **Figure 2** illustrates the general process by which γ-secretase acts on the substrates to exert important functions. The γ-secretase substrates associated with cancer mainly include Notch, ErbB4, CD44, Cadherins, VEGFR1, IGF1R, MUC1, etc. They are involved in various cellular pathways, including the regulation of cell fate, transcriptional regulation, cell adhesion, and neurotrophin signal transduction 26.

**Notch:** As a transmembrane receptor protein, Notch is one of the main targets of γ-secretase activity. It is a cell fate sensor that serves as a receptor for a variety of classical and non-classical ligands (such as Delta, Jagged), and upon binding, Notch is cleaved by γ-secretase and releases an active ICD (here called NICD). This allows NICD to transfer to the nucleus, where it binds to the transcription factor CSL and MAML, which in turn activates downstream effectors (such as Hes1), preventing irreversible cell differentiation and cell cycle exit 27-29. There are four members of the human Notch receptor family, namely Notch 1, Notch 2, Notch 3, and Notch 4, whose ligands are also transmembrane proteins including Delta-like-1, Delta-like-2, Delta-like-3, Delta-like-4, Jagged-1, and Jagged-2 30. The Notch signaling pathway is an evolutionarily conserved pathway whose dysregulation has been implicated in various human cancers 31. Current data 32, 33 also confirm that the oncogenic range of Notch signaling is partly due to its crosstalk with other signaling pathways, such as NF-kB, Hedgehog, JAK/STAT, MAPK, HIF-1α, Wnt, TGF-p, VEGF, PI3K/Akt, Ras, etc. In fact, based on the overwhelming evidence for the role of Notch signaling in cancer, this pathway has clearly become an important target for cancer therapy.

**CD44:** CD44, a non-kinase transmembrane glycoprotein involved in cell-cell interactions, is a receptor for hyaluronic acid and is overexpressed in a variety of cell types, including cancer stem cells 34, 35. It is a major adhesion molecule and can also activate cell signal pathways, and mediate various biological processes, including lymphocyte homing, cell proliferation, migration and metastasis 36, 37. CD44 can release the active intracellular domain (CD44-ICD) upon cleavage of γ-secretase 38, 39, which can translocate to the nucleus and trigger downstream signaling pathway. Overall, as an important cell surface adhesion molecule, the association of CD44 with human malignancies has been extensively reported.

**ErbB4:** ErbB4 is a type I transmembrane receptor tyrosine kinase that binds to its ligand (heregulin) and is cleaved by metalloproteinases to produce ECD containing transmembrane and cytoplasmic domains. The ECD is then cleaved by γ-secretase and releases the cytoplasmic domain into the cytoplasm, thereby regulating cell proliferation and differentiation 40, 41. Previous studies 42, 43 have also confirmed the important role of ErbB4 in cancer.

**E-cadherins and N-cadherins:** Cadherins, a class of cell adhesion molecules, are essential for maintaining cell-cell contact and for regulating cytoskeletal complexes 44, 45. Cadherins rely on calcium ions (Ca^2+^) to function. And they regulate the development and function of most tissues and have important roles in cell signaling, proliferation, and differentiation. Epithelial cadherins (E-cadherins) bind to PS1 and are treated by γ-secretase 46. Neurocadherins (N-cadherins) experience PS1-mediated cleavage of γ-secretase to produce ICDs, which are potent repressor of the cAMP response element binding protein (CREB) and CREB binding protein (CBP)-mediated transcription 46, 47. E-cadherins and N-cadherins play a key role in maintaining the structural integrity and polarity of epithelial tissues, and their close relationship with tumor progression as important markers of epithelial mesenchymal transformation (EMT) has been demonstrated by numerous studies.

Overall, many γ-secretase substrates are closely related to the occurrence and development of cancer. Here we list only a few substrates that are widely reported and relatively well studied. Other substrates, such as VEGFR, IGF1R, and MUC1, have also been shown to be associated with neoangiogenesis and cell adhesion, and γ-secretase may also regulate tumorigenesis by affecting these effects of the above substrates.

## The correlation between components of γ-secretase and cancer

At present, related studies on γ-secretase have mainly focused on the function of substrates or the role of inhibitors/modulators as well as spatial structure of γ-secretase. However, the potential role of individual γ-secretase components, including potential links to cancer, has not been fully understood. Here, we pool the forefront data from γ-secretase studies to attempt to answer this question.

**PS:** PS is the major component of the γ-secretase complex. It is so named because its mutation is closely related to early-onset familial inherited AD and belongs to the member of the evolutionary conserved gene family. PS (PS-1 or PS-2) can be encoded by PSEN1 or PSEN2. PSEN1 is the most commonly mutated gene in patients with familial inherited AD, accounting for 70% to 80%. While mutations in PSEN2 gene are rare 48. PSEN2 has been reported to play an important role in promoting the progression of lung tumors 49 and gliomas 50. However, studies on the relationship between PSEN2 and cancer are still lacking, and whether PSEN2 plays similar roles in other tumors is still unknown.

As the highly homologous gene of PSEN2, PSEN1 has been shown to be associated with various tumorigenic processes, such as cell proliferation, apoptosis, and cell adhesion 51, 52. As the core catalytic subunit of the γ-secretase complex, PSEN1 can generate activated Notch intracellular domain (NICD) by cleaving Notch family proteins, and then translocate NICD to the nucleus to regulate the transcriptional expression of a series of target genes. Besides, it interacts with Wnt/β-catenin, PI3K/AKT/mTOR and RAS/RAF/MEK pathways 53, 54, and is involved in cell proliferation, invasion, metastasis and neovasculangiogenesis of malignant tumors. Similarly, CD44, cadherins, as important transmembrane proteins, can also participate in their intracellular signal transduction through a similar mechanism to regulate the biological behaviors of tumor cells, thus affecting the progression of tumor 38, 39.

Additionally, as the catalytic core of γ-secretase complex, the expression level and mutation of PSEN1 obviously directly affect the activity and mode of action of γ-secretase. Importantly, γ-secretase has many substrates, and there are many signaling pathways affected by it. Thus, this undoubtedly highlights the broad and complex roles of PSEN1. It should also be noted that PSEN1 plays an important role in various cancers, both dependent on and independent of γ-secretase activity. For example, PSEN1/γ-secretase can, on the one hand, generate activated Notch-ICD and CD44-ICD by cutting Notch and CD44, translocate them to the nucleus, and bind with its activation transcription factors to activate downstream target genes. On the other hand, it can act on β-catenin independently of γ-secretase activity to activate the WNT signaling pathway 53, 55.

Although most genes involved in tumorigenesis can be divided into tumor suppressor genes and oncogenes, PSEN1 cannot be clearly classified, because it exhibits two functions of promoting and suppressing cancer in different tumor-specific genetic damage. A previous study 56 found that PSEN1 expression was significantly up-regulated in both head and neck squamous cell carcinoma (HNSCC) cell lines and tissue samples, and was associated with poor prognosis and radiotherapy resistance in HNSCC. Similar findings were also seen in hepatocellular carcinoma 57 and oesophageal cancer 58. In addition, the down-regulation of PSEN1 expression in cell line U937 led to slower proliferation and increased apoptosis of tumor cells, and the down-regulation of PSEN1 expression also reduced the tumor-causing ability in nude mice 59. These studies suggest that PSEN1 plays a “driving” role in some cancer diseases, acting as an oncogene. However, other studies have found that PSEN1 plays an opposite role in some tumors. For example, in glioblastoma, PSEN1 can inhibit tumor cell invasiveness 60. Additionally, Xia et al. 61 found that PSEN1 knockout mice spontaneously formed skin malignations due to the absence of PSEN1, which leads to the accumulation of β-catenin in the cytoplasm and nucleus, thus activating the β-catenin signaling pathway and resulting in increased cyclin D1 expression. It was also shown that increased expression of PSEN1 was associated with good disease-free survival in patients with breast cancer 62, suggesting that PSEN1 might play a role in inhibiting the formation and progression of some tumors. To sum up, it is not difficult to find that PSEN1 plays different or even opposite roles in different cancer diseases, which may be related to the tissue-specific microenvironment in which different cancers occur.

**Pen-2/PSENEN:** Pen-2/PSENEN is the minimal subunit of the γ-secretase complex. Current data indicate that PSENEN is involved in the occurrence and development of many human diseases, such as hidradenitis suppurative (HS) and Dowling's disease (DDD). PSENEN has also been found to play a vital role in adipocyte differentiation 63. Moreover, PSENEN deletion can inhibit HES1 and activate STAT3 to trigger GFAP activation, thereby promoting the differentiation of oligodendrocyte progenitors into astrocytes 64. Up to now, the study of PSENEN in cancer is still scarce, and the only studies are mostly stuck in bioinformatics analysis and lack of in-depth exploration of wet experiments. Gu et al. 65 found that the expression of PSENEN was increased in low-grade gliomas based on bioinformatics methods, which was corresponding to the poor prognosis of patients. Similarly, a similar phenomenon was observed in pancreatic cancer 66, suggesting that PSENEN may play a “bad” role as an oncogene in some tumors. However, based on the same bioinformatics analysis, Chen et al. 67 found that PSENEN was downregulated in gastric cancer tissues, and its low expression level was associated with worse prognosis. This seems to suggest that PSENEN as a tumor suppressor plays a “good” role in gastric cancer. Notably, these findings are still unsupported by adequate evidence. The specific role of PSENEN in different human cancers still needs further study.

**Nicastrin/NCSTN:** NCSTN is the largest subunit of the γ-secretase complex, with a single TMD and a large ECD. These domains were identified as functional sites for the recruitment of γ-secretase substrates 68. NCSTN is mainly synthesized by fibroblasts and neurons, and is widely distributed in the body. A growing number of studies have reported a close relationship between NCSTN and tumorigenesis and progression. NCSTN was highly expressed in breast cancer and had carcinogenic effects 69, 70. Overexpression of NCSTN can regulate the properties of breast cancer stem cells and induce the epithelial-mesenchymal transition (EMT) by cleaving the Notch1 protein 69-71. Moreover, NCSTN can also regulate AKT activation in hepatocellular carcinoma, which in turn affects cellular malignant behaviors 72. The process by which NCSTN controls cell death through the PI3K/Akt pathway is independent of γ-secretase, that is, NCSTN can independently perform some functions 73, 74. Filipovic A et al. 74 found that specific monoclonal antibodies against NCSTN had anti-tumor effects on invasive triple-negative breast cancer cells. Furthermore, siRNA-NCSTN has been found to prevent the induction of the Notch1 intracellular domain (NICD) after oxaliplatin, thereby affecting the response to chemotherapy in colon cancer 75. Another study also demonstrated that siRNA-NCSTN in basal-like breast cancer could enhance the anti-tumor effect of EGFR inhibitors by blocking the Notch and AKT signaling pathways 76. Taken all together, NCSTN does participate in the occurrence and progression of some tumors. However, on the whole, the study of NCSTN in tumor is still insufficient, and a lot of research is needed to reveal the more specific roles of NCSTN in tumor.

**Aph-1:** Increasing evidence indicates that γ-secretase plays a critical role in cancer development and progression. Although Aph-1, as an important member of the γ-secretase complex, is important in performing biological processes such as cleaving transmembrane proteins, there is still a significant lack of research on Aph-1 in cancers. Human Aph-1 is encoded by two genes, APH1A and APH1B, of which APH1A appears to be more important 77. APH1A was overexpressed in diffuse large B-cell lymphoma with poor prognosis 78, and its expression level was also positively correlated with the grade of hepatocellular carcinoma 79. Peltonen HM et al. 80 investigated the expression levels of γ-secretase subunits in breast cancer, and found that the mRNA expression of APH1B, PSENEN, and NCSTN was significantly reduced in breast cancer cases with higher tumor grade. In addition, APH1B was also reported to be involved in the maintenance of spherical cell stemness in cervical cancer 81. Importantly, although these studies have observed some association of Aph-1 with some tumors, the specific mechanism remains poorly investigated. The role of Aph-1 as an important component of γ-secretase in cancer occurrence and progression remains to be uncovered.

## The potential and challenges of γ-secretase inhibitors as anticancer therapeutic strategies

In the previous section, we have discussed the association of substrates and each component of γ-secretase with cancer. The intriguing question is whether targeting γ-secretase can be an effective anticancer strategy, given its close association with cancer. In fact, γ-secretases have been proposed as therapeutic targets for human diseases, including cancer. Two candidates for targeting the γ-secretase complex have emerged: inhibitors and modulators. γ-secretase inhibitors (GSIs) were originally developed as a treatment for AD. However, to date, GSIs has not gained a good indication for the treatment of AD. This is well illustrated by the adverse reactions of Semagacestat (a broad-spectrum GSI), in the Phase III clinical trial of AD 82. GSIs has been repurposing as an anticancer drug, due to its ability to block γ-secretase activity and inhibit Notch cleavage. The anti-tumor effects of GSIs in various cancers have been extensively studied. In **Table 1**, we summarize the GSIs and their associated information in the preclinical models. Globally, the anti-cancer efficacy of GSIs in preclinical models is promising, which is believed that GSIs can drive tumor cell differentiation and apoptosis through multiple mechanisms, reduce the burden of cancer stem cells, and also hinder EMT, and overcome resistance to conventional therapies. Disappointingly, however, these GSIs have not performed well in clinical trials. Because most solid tumors do not derive clinical benefit from them. J.Bart Rose and Tyler R.McCaw et al. 26 reviewed the relevant information of clinical research of GSIs in various cancer types in detail. Here, we update and sort out this data (**Table 2**). It can be rationally seen, that although GSIs do not confer significant clinical activity in the majority of patients in some cancer types, it is undeniable that GSIs do show anti-cancer effects in some patients. For example, in a phase II clinical trial of RO4929097 monotherapy for metastatic refractory pancreatic cancer 83, 3 of 12 (25%) evaluable patients had stable disease, while the other 9 patients did not benefit from RO4929097. In another phase I trial of MK-0752 in adult patients with advanced solid tumors, 5 of the 21 evaluable glioma patients showed clinical benefit, although none of the patients with other tumors (breast cancer, colorectal cancer, ovarian cancer, sarcoma cancer, etc.) gained clinical benefit 84. Encouragingly, a recently published Phase 3 international, double-blind, randomized, placebo-controlled trial of nirogacestat (a potent, orally active, reversible, non-competitive and selective GSI) for progressive desmoid tumors has yielded exciting results 85. Globally, the therapeutic efficacy of GSIs varies by cancer type and individual patient. The reasons responsible for the apparently different clinical activities of GSIs in patients of different cancer types as well as in different patients of the same cancer type are diverse and complex. In addition to individual heterogeneity among patients, the dose and duration of GSIs, as well as the treatment strategies of patients prior to this treatment may be important reasons for this difference.

According to the currently available data, the mechanism of anti-tumor action of GSIs is still elusive. Since many γ-secretase substrates are directly involved in carcinogenesis or tumor progression, GSIs should theoretically be an ideal anti-cancer strategy. However, this is not the case (as noted above). These disappointing clinical manifestations reflect important issues that still deserve our consideration. The first is the non-selective suppression of GSIs. γ-secretase as a protease complex can cleave a variety of transmembrane proteins with different biological functions, which adds diversity and complexity to the biological pathways in which γ-secretases participate. Non-selective inhibition of this complex is bound to cause off-target toxicity, which may be refractory.

A typical example is that the GSIs currently used for anti-cancer therapy works primarily by blocking Notch signaling. However, the impact of Notch signaling on the body is comprehensive and profound, and in addition to its impact on cancer cells, it is also critical to the fate of various cells during embryonic development and adulthood. In addition to counteracting the pro-cancer effects of Notch signaling, GSIs may also affect other important signaling and cellular events, leading to unacceptable toxic effects. Of course, we should also note that some types of cancer cells do not rely excessively on Notch signaling for survival. Conversely, activation of Notch signaling even blocks the formation of some malignancies 86. Aaron Proweller et al also reported that impaired Notch signaling promoted the formation of nascent squamous cell carcinoma 87. Another question worth considering is why the success of GSIs in preclinical research is difficult to translate in clinical trials. Going back to the essential differences between preclinical research and clinical trials, this phenomenon is not difficult to explain. First, preclinical studies are largely conducted on various cell lines, which are in vitro experiments that lack the involvement of the body's immune metabolic processes. Moreover, animal models in preclinical studies are unable to fully mimic the pathophysiological features of the human body, and even some animal models are not immunocompetent. It is worth noting that, although GSIs may hinder tumor progression through Notch signaling, at the same time it may in turn impair the body's anti-tumor immune response. LI-CHO (a GSI) has been reported to inhibit the proliferation of mouse CD8 T cells (a major effector of anti-tumor immunity) in a dose-dependent manner 88. While the expression of effector molecules in CD8 T cells also requires the triggering of Notch signaling 89. As highlighted by J.Art Rose and Tyler R. McCaw et al. 26, the large number of immunosuppressive cells in the tumor microenvironment can also be a great challenge. Charbonnier LM et al. 90 found that Notch signaling can disrupt the stability of Treg, and GSIs as a blocker of Notch signaling may promote Treg-mediated immunosuppression and impair anti-tumor immunity. The mechanism by which GSIs weaken the body's anti-tumor immunity may be much more than that, and further investigations are pending.

The implementation of GSIs may require individualized and targeted management. Addressing the current unfavorable situation of GSIs requires a better understanding of the fine structure and specific mechanisms of γ-secretases, which may facilitate the development of more efficient and selective GSIs. At the same time, the optimization of GSIs anticancer strategy should also include the dose, frequency and duration of drug use, as well as the rational combination strategy with other drugs.

## Summary and outlook

As a multisubstrate protease complex, γ-secretase is involved in several biological pathways of the organism. Among the substrates of γ-secretase action, many are closely associated with the occurrence and progression of cancer, of which Notch receptor is the most concerned. Given the promoting role of Notch in multiple cancers, GSIs have also been developed in anticancer therapy. However, it is frustrating that the success of GSIs in preclinical models is not replicated in clinical trials. Although there has been significant progress in the understanding of γ-secretase (especially the three-dimensional structure), there is no denying that we still do not fully understand it. Several key issues still remain to be elucidated. For example, what are the specific biological mechanisms by which γ-secretase acts on substrates? Is the effect of GSIs on anti-tumor immunity related to cancer context? Since GSIs can somehow affect multiple receptors and block several key signaling pathways, is it necessary to target the interference of these non-destination signals simultaneously? Further dissecting and revealing the complexity of γ-secretase structure and function, as well as selecting better diseased animal models to track the multidimensional dynamic effects of γ-secretase in vivo, should be the direction of future efforts. Here, we systematically review the important components of γ-secretases and their relevance to cancer, focusing on combing and discussing the potential and problems of current GSIs in cancer treatment. Taken together recent breakthrough findings in the field of γ-secretase research, we believe that despite the unsatisfactory performance of GSIs in clinical trials, after overcoming these challenges, GSIs will remain a promising strategy for anticancer therapy.

## Figures and Tables

**Table 1:** Performance of GSIs in preclinical models.

GSIs	Cancer Types	Intervention objects	Effect	Reference
GSI and LY-411,575	Kaposi's sarcoma	Kaposi's sarcoma tumor cells	Induction of apoptosis	PMID: 15940249 91
GSI-XII (Z-IL-CHO) and GSI-IX (DAPT)	Multiple myeloma	NCI-H929, U266 and RPMI-8226	Induction of apoptosis	PMID: 21965140 92
Z-LLNle-CHO	Breast cancer	MCF-7, BT474, T47D, SKBR3, MDA-MB-231, and MDA-MB-468	Z-LLNle-CHO mediates the damage to breast cancer cells through proteasome inhibition (rather than γ -secretase inhibition)	PMID: 19660128 93
LLNle	Glioblastoma	human glioblastoma tumor-initiating cells (GBM TICs)	LLNle mediates the GBM TICs apoptotic cell death through γ-secretase and proteasome inhibition	PMID: 19861404 94
MRK-003	Pancreatic cancer	Pa03C, Pa14C, Pa16C and Pa29C; patient-derived PDAC xenografts	MRK-003 can reduce tumor cell proliferation, induce apoptosis and intratumoral necrosis	PMID: 22752426 95
DAPT	Ovarian cancer	SKOV3 and HO8910	DAPT prevents ovarian cancer stem cells (OCSCs) formation, and inhibits OCSC self-renewal and proliferation	PMID: 23482909 96
RO4929097	Melanoma	WM35, WM98.1, WM115, WM983A, WM3248, A375, WM239A/131/4-5B1 (5B1); human primary melanoma xenograft in NOD/SCID/IL2gammaR-/- mice	RO4929097 can weaken cell proliferation and tumor growth of Melanoma.	PMID: 21980408 97
MRK-003, MRK-006	T-cell acute lymphoblastic leukemias (T-ALL)	T-All cells	Combination of GSI with a CDK4 inhibitor results in potent cell cycle arrest and death.	PMID: 19318552 98
RO4929097	NSCLC	A549, H460a cells, A549 NSCLC xenograft model	Significant tumor growth inhibition	PMID: 19773430 99
PF-03084014	Prostate Cancer	Du145, PC3 and Du145R, PC3R; 7-8-week-old male NOD.CB17-Prkdcscid/NCrCrl (NOD/SCID) mice	PF-03084014 enhanced the docetaxel-mediated tumor response	PMID: 26202948 100
MK-0725	Ovarian cancer	A2780, OVCAR3, SKOV3, HO8910PM; Mouse xenograft model of A2780	Induction of apoptosis; Significant tumor growth inhibition	PMID: 26704638 101
GSI I and GSI XX	NSCLC	H460, A549 and H1395	Treatment with GSIs after radiation can significantly enhance radiation-mediated tumour cytotoxicity and delay tumor progression.	PMID: 22596234 102
DAPT	Ovarian cancer	A2780, A2780/CP70 and OV2008, OV2008/C13	DAPT pretreatment can improve the sensitivity of cisplatin-resistant human ovarian cancer cells to cisplatin.	PMID: 24535252 103
BMS-708163	Lung Cancer	PC9, PC9/AB2, PC9/AB2 xenografts	BMS-708163 can sensitize PC9/AB2 cells to gefitinib-induced cytotoxicity. BMS-708163 combined with gefitinib can induce high level of apoptosis. And the combination of gefitinib and BMS-708163 can inhibit the growth of PC9/AB2 xenografts.	PMID: 25561332 104
GSI I (cbz-IL-CHO)	Gastric cancer	AGS, SNU601, SNU638, SNU-668, SNU-719, MKN28, and YCC-2; orthotopically transplanted gastric cancer mouse models	GSI I can significantly inhibit the proliferation of gastric cancer cells and reduce the tumor load of orthotopic transplantation mouse models, and the combination of GSI I and 5-FU can enhance the therapeutic effect	PMID: 26134677 105

**Table 2:** Performance of GSIs as anti-cancer strategies in clinical trials.

Cancer Types	Name	Phase	Case selection	Usage	Outcome	Reference
Lung cancer	MK-0752	I	Age ≥ 18 years; patients with histologically confirmed solid tumors that had failed to respond to standard therapies or for which no proven treatments existed.	Oral administration; Specific dose and time are not given (Despite providing the drug dose and duration of use in each schedule, the specific cancer type was not specified);	None of the patients received any clinical benefit (n=3).	PMID: 22547604 84
	PF-03084014	I	Advanced patients who were resistant to standard therapy or for which no therapy was available.	Oral administration for 21 days in a test dose range of 20 to 330 mg BID (Please refer to this article for specific usage);	None of the patients received any clinical benefit (n=5).	PMID: 25231399 106
	LY900009	I	Age ≥ 18 years; patients with advanced cancer refractory to standard therapy (or no available standard therapy) and a 12-week expectancy.	LY900009 was administered orally thrice weekly (Monday, Wednesday, and Friday) on a 28-d cycle; Dose escalation was performed at a pre-specified dose level (2 - 60 mg);	Of the 2 patients evaluable for response, 1 patient was observed with SD (73d).	PMID: 26798966 107
	RO4929097 + Cediranib	I	Age ≥ 18 years; Patients had histologically or cytologically documented advanced solid malignancy, refractory to standard therapy or for which conventional therapy was not effective.	Patients received a progressively increased dose of RO4929097 (on a 3 days-on and 4 days-off schedule) in combination with cediranib (once daily). The first cycle, which lasted 42 days, was given RO4929097 alone for the first 3 weeks, followed by RO4929097 and cediranib in combination from day 22. The second and subsequent periods were 21 days.	None of the patients received any clinical benefit (n=1).	PMID: 23868004 108
Pancreatic cancer	MK-0752	I	Age ≥ 18 years; patients with histologically confirmed solid tumors that had failed to respond to standard therapies or for which no proven treatments existed.	Oral administration; Specific dose and time are not given (Despite providing the drug dose and duration of use in each schedule, the specific cancer type was not specified).	None of the patients received any clinical benefit (n=2).	PMID: 22547604 84
	LY900009	I	Age ≥ 18 years; patients with advanced cancer refractory to standard therapy (or no available standard therapy) and a 12-week expectancy.	LY900009 was administered orally thrice weekly (Monday, Wednesday, and Friday) on a 28-d cycle; Dose escalation was performed at a pre-specified dose level (2-60 mg).	None of the patients received any clinical benefit (n=3).	PMID: 26798966 107
	PF-03084014	I	Advanced patients who were resistant to standard therapy or for which no therapy was available.	Oral administration for 21 days in a test dose range of 20 to 330 mg BID (Please refer to this article for specific usage);	None of the patients received any clinical benefit (n=2).	PMID: 25231399 106
	RO4929097	II	Age ≥ 18 years; patients with previously treated metastatic pancreatic adenocarcinoma.	Oral administration; 20 mg daily on days 1-3, 8-10 and 15-17 of 21-day cycles;	Three (25%) of 12 evaluable patients achieved stable disease. Median PFS was 1.5 months.	PMID: 24668033 83
	R04929097 + Gemcitabine	II	Patient was 18 years or older, had histologically or cytologically proven advanced solid tumors with no further standard treatment options available.	RO4929097 was administered orally, once daily on days 1-3, 8-10, 15-17, 22-24. RO4929097 dose levels were 20 mg, 30 mg, 45 mg and 90 mg; Gemcitabine was administered at 1000 mg/m^2^ on d1, 8, and 15 in 28 d cycles.	One in three patients with pancreatic cancer achieved long-term stable disease (> 4 months).	PMID: 23645447 109
	MK-0752 + Gemcitabine	I	Patients with stage III (inoperable) and stage IV pancreatic ductal adenocarcinoma (Of the 44 patients included, 93% had stage IV pancreatic cancer, and 30% had received prior chemotherapy)	MK-0752 was administered orally weekly; Gemcitabine was administered intravenously at 800 or 1000 mg m-2 on days 1,8, and 15 (28-day cycles).	Of the 19 patients undergoing response assessment, one confirmed partial response and 13 had stable disease.	PMID: 29438372 110
Melanoma	RO4929097	I	Age ≥ 18 years; patients with histologically confirmed solid tumors refractory to standard therapy or for which no standard therapy exists.	Oral increasing doses of RO4929097 by two regimens: (A) 3 consecutive days per week for 2 weeks every 3 weeks; (B) 7 consecutive days every 3 weeks.	Of the 24 melanoma patients with evaluable efficacy, one nearly complete FDG-PET response.	PMID: 22529266 111
	RO4929097	II	Patient had stage IV melanoma of histologically confirmed skin or unknown origin (excluding ocular and mucosal sources), had not received chemotherapy (immunotherapy and adjuvant therapy were allowed) and had no history of central nervous system metastasis.	Taken orally on an empty stomach at a dose of 20 mg daily 3 consecutive days per week.	Of the 32 evaluable patients, 1 confirmed partial response persisted for 7 months and 8 patients had stable disease until at least week 12, with 1 continuing for 31 months.	PMID: 25250858 112
	MK-0752	I	Age ≥ 18 years; patients with histologically confirmed solid tumors that had failed to respond to standard therapies or for which no proven treatments existed.	Oral administration; Specific dose and time are not given (Despite providing the drug dose and duration of use in each schedule, the specific cancer type was not specified)	None of the patients received any clinical benefit (n=3).	PMID: 22547604 84
	LY3039478	I	Age ≥ 20 years; Japanese patients with advanced solid tumors for whom standard therapies failed or would not be appropriate.	2 dose levels of crenigacestat (25 mg and 50 mg) were administered orally 3 times weekly (TIW) over a 28-day cycle.	None of the patients received any clinical benefit (n=2).	PMID: 32939607 113
Ovarian cancer	RO4929097	I	Age ≥ 18 years; patients with histologically confirmed solid tumors refractory to standard therapy or for which no standard therapy exists.	Oral increasing doses of RO4929097 by two regimens: (A) 3 consecutive days per week for 2 weeks every 3 weeks; (B) 7 consecutive days every 3 weeks.	Of the 9 ovarian cancer patients with evaluable efficacy, 0 showed clinical benefit.	PMID: 22529266 111
	LY900009	I	Age ≥ 18 years; patients with advanced cancer refractory to standard therapy (or no available standard therapy) and a 12-week expectancy.	LY900009 was administered orally thrice weekly (Monday, Wednesday, and Friday) on a 28-d cycle; Dose escalation was performed at a pre-specified dose level (2-60 mg).	None of the patients received any clinical benefit (n=11).	PMID: 26798966 107
	RO4929097	II	Age ≥ 18 years; Women with progressive platinum-resistant epithelial ovarian cancer treated with ≤ 2 chemotherapy regimens for recurrent disease;	RO4929097 administered orally at 20 mg once daily, 3 days on/4 days off each week in a three-week cycle.	No objective responses were observed. 15 patients (33%) had SD as their best response, with a median duration of 3.1 months.	PMID: 25769658 114
	MK-0752	I	Age ≥ 18 years; patients with histologically confirmed solid tumors that had failed to respond to standard therapies or for which no proven treatments existed.	Oral administration; Specific dose and time are not given (Despite providing the drug dose and duration of use in each schedule, the specific cancer type was not specified)	None of the patients received any clinical benefit (n=3).	PMID: 22547604 84
	R04929097 + Gemcitabine	II	Patient was 18 years or older, had histologically or cytologically proven advanced solid tumors with no further standard treatment options available.	RO4929097 was administered orally, once daily on days 1-3, 8-10, 15-17, 22-24. RO4929097 dose levels were 20 mg, 30 mg, 45 mg and 90 mg; Gemcitabine was administered at 1000 mg/m^2^ on d1, 8, and 15 in 28 d cycles.	None of the patients received any clinical benefit (n=2).	PMID: 23645447 109
	RO4929097 + Temsirolimus	Ib	Age ≥ 18 years; patients with histologically confirmed advanced, incurable solid malignancy refractory to conventional therapy or for which no standard therapy existed.	RO4929097 and Temsirolimus were given in three progressively incremented dose levels. RO4929097 was orally administered on an empty stomach on a 3 days on/4 days off schedule, weekly; Intravenous temsirolimus every week.	No objective responses were observed.	PMID: 23860641 115
	RO4929097 + Cediranib	I	Age ≥ 18 years; Patients had histologically or cytologically documented advanced solid malignancy, refractory to standard therapy or for which conventional therapy was not effective.	Patients received a progressively increased dose of RO4929097 (on a 3 days-on and 4 days-off schedule) in combination with cediranib (once daily). The first cycle, which lasted 42 days, was given RO4929097 alone for the first 3 weeks, followed by RO4929097 and cediranib in combination from day 22. The second and subsequent periods were 21 days.	None of the patients received any clinical benefit (n=1).	PMID: 23868004 108
Sarcoma	RO4929097	I	Age ≥ 18 years; patients with histologically confirmed solid tumors refractory to standard therapy or for which no standard therapy exists.	Oral increasing doses of RO4929097 by two regimens: (A) 3 consecutive days per week for 2 weeks every 3 weeks; (B) 7 consecutive days every 3 weeks.	Of the 12 sarcoma patients with evaluable efficacy, 1 showed mixed response (stable disease).	PMID: 22529266 111
	MK-0752	I	Age ≥ 18 years; patients with histologically confirmed solid tumors that had failed to respond to standard therapies or for which no proven treatments existed.	Oral administration; Specific dose and time are not given (Despite providing the drug dose and duration of use in each schedule, the specific cancer type was not specified)	None of the patients received any clinical benefit (n=3).	PMID: 22547604 84
	LY900009	I	Age ≥ 18 years; patients with advanced cancer refractory to standard therapy (or no available standard therapy) and a 12-week expectancy.	LY900009 was administered orally thrice weekly (Monday, Wednesday, and Friday) on a 28-d cycle; Dose escalation was performed at a pre-specified dose level (2-60 mg).	Of the 2 patients evaluable for response, 1 patient was observed with SD (113d).	PMID: 26798966 107
Glioma	MK-0752	I	Patients, aged between 3 and 21 years, were histologically proven malignant central nervous system tumor (diffuse pontine glioma does not require histology) and refractory to conventional therapy with Lansky or Karnofsky score 60.	MK-0752 was taken orally in a starting dose of 200 mg/m^2^ once every 7 days for three consecutive days.	Most patients experienced disease progression after 1 or 2 courses. Prolonged SD was observed only in 2 patients (≥3 courses).	PMID: 21825264 116
	RO4929097 +Bevacizumab	I	Patient was 18 years or older, had histologically proven malignant glioma, and progressed after radiotherapy and chemotherapy with temozolomide.	RO4929097 was taken orally for 3 days on/4 days off each week for 4 consecutive cycles (days 1-3, 8-10, 15-17, and 22-24), and intravenous infusion of bevacizumab (Day 1 and Day 15, 10mg /kg) every 2 weeks.	Two of the 12 patients had radiological responses (one patient gained CR and the other PR).	PMID: 27826680 117
	MK-0752	I	Age ≥ 18 years; patients with histologically confirmed solid tumors that had failed to respond to standard therapies or for which no proven treatments existed.	Oral administration; Specific dose and time are not given (Despite providing the drug dose and duration of use in each schedule, the specific cancer type was not specified)	Of the 21 patients with evaluable efficacy, 5 showed SD.	PMID: 22547604 84
Breast cancer	MK-0752	I	Age ≥ 18 years; patients with histologically confirmed solid tumors that had failed to respond to standard therapies or for which no proven treatments existed.	Oral administration; Specific dose and time are not given (Despite providing the drug dose and duration of use in each schedule, the specific cancer type was not specified)	None of the patients received any clinical benefit (n=24).	PMID: 22547604 84
	RO4929097	I	Age ≥ 18 years; patients with histologically confirmed solid tumors refractory to standard therapy or for which no standard therapy exists.	Oral increasing doses of RO4929097 by two regimens: (A) 3 consecutive days per week for 2 weeks every 3 weeks; (B) 7 consecutive days every 3 weeks.	None of the patients received any clinical benefit (n=10).	PMID: 22529266 111
	PF-03084014	I	Advanced patients who were resistant to standard therapy or for which no therapy was available.	Oral administration for 21 days in a test dose range of 20 to 330 mg BID (Please refer to this article for specific usage);	None of the patients received any clinical benefit (n=7).	PMID: 25231399 106
	RO4929097 + Exemestane	Ib	Patients with ER+/HER2- metastatic breast cancer	RO4929097 was taken orally every day for 3 consecutive days, followed by 4 days of discontinuation, and the cycle was 21 days. Exemestane was used at a dose of 25 mg daily.	Of the 14 evaluable patients, 8 patients showed clinical responses (1 PR and 7 SD). The overall clinical benefit rate (CR + PR + SD >= 6 months) was 20% and PFS was 3.2 months.	PMID: 34903452 118
	R04929097 + Gemcitabine	II	Patient was 18 years or older, had histologically or cytologically proven advanced solid tumors with no further standard treatment options available.	RO4929097 was administered orally, once daily on days 1-3, 8-10, 15-17, 22-24. RO4929097 dose levels were 20 mg, 30 mg, 45 mg and 90 mg; Gemcitabine was administered at 1000 mg/m^2^ on d1, 8, and 15 in 28 d cycles.	Of the 5 patients, 1 patient showed SD (> 4 months).	PMID: 23645447 109
	PF-03084014 + Docetaxel	I	Adult women with advanced or metastatic triple-negative breast cancer or hormone-refractory ER/PR-positive breast cancer.	PF-03084014 was taken orally twice daily continuously in combination with intravenous docetaxel given on day 1 of each 21-day cycle.	4 of the 25 evaluable patients achieved a confirmed partial response. 9 (36%) patients had stable disease, 5 of whom had unconfirmed partial responses. 11 (44%) patients had the best overall response to progressive disease. The median PFS was 4.1 months, and the 6-month PFS rate was 17.1%.	PMID: 27906684 119
	MK-0752 + Docetaxel	Ib	Male or female patients with advanced breast cancer not responsive to first-line anthracycline chemotherapy.	MK-0752 was used on days 1 to 3, with the dose determined by a dose-escalation protocol, followed by docetaxel on day 8 of each 21-day cycle.	Of the 24 patients evaluable for response, 11 patients were observed with PR, 9 SD, and 3 PD.	PMID: 23340294 120
	RO4929097 + Cediranib	I	Age ≥ 18 years; Patients had histologically or cytologically documented advanced solid malignancy, refractory to standard therapy or for which conventional therapy was not effective.	Patients received a progressively increased dose of RO4929097 (on a 3 days-on and 4 days-off schedule) in combination with cediranib (once daily). The first cycle, which lasted 42 days, was given RO4929097 alone for the first 3 weeks, followed by RO4929097 and cediranib in combination from day 22. The second and subsequent periods were 21 days.	None of the patients received any clinical benefit (n=1).	PMID: 23868004 108
Colorectal cancer	MK-0752	I	Age ≥ 18 years; patients with histologically confirmed solid tumors that had failed to respond to standard therapies or for which no proven treatments existed.	Oral administration; Specific dose and time are not given (Despite providing the drug dose and duration of use in each schedule, the specific cancer type was not specified)	None of the patients received any clinical benefit (n=16).	PMID: 22547604 84
	PF-03084014	I	Advanced patients who were resistant to standard therapy or for which no therapy was available.	Oral administration for 21 days in a test dose range of 20 to 330 mg BID (Please refer to this article for specific usage);	None of the patients received any clinical benefit (n=11).	PMID: 25231399 106
	LY900009	I	Age ≥ 18 years; patients with advanced cancer refractory to standard therapy (or no available standard therapy) and a 12-week expectancy.	LY900009 was administered orally thrice weekly (Monday, Wednesday, and Friday) on a 28-d cycle; Dose escalation was performed at a pre-specified dose level (2-60 mg).	Of the 5 patients evaluable for response, 1 patient (rectal carcinoma) was observed with SD (55d).	PMID: 26798966 107
	RO4929097 + Cediranib	I	Age ≥ 18 years; Patients had histologically or cytologically documented advanced solid malignancy, refractory to standard therapy or for which conventional therapy was not effective.	Patients received a progressively increased dose of RO4929097 (on a 3 days-on and 4 days-off schedule) in combination with cediranib (once daily). The first cycle, which lasted 42 days, was given RO4929097 alone for the first 3 weeks, followed by RO4929097 and cediranib in combination from day 22. The second and subsequent periods were 21 days.	Of the 6 patients evaluable for response, 2 patients were observed with SD (7 and 11 cycles).	PMID: 23868004 108
	RO4929097	I	Age ≥ 18 years; patients with histologically confirmed solid tumors refractory to standard therapy or for which no standard therapy exists.	Oral increasing doses of RO4929097 by two regimens: (A) 3 consecutive days per week for 2 weeks every 3 weeks; (B) 7 consecutive days every 3 weeks.	None of the patients received any clinical benefit (n=12).	PMID: 22529266 111
	LY3039478	I	Age ≥ 20 years; Japanese patients with advanced solid tumors for whom standard therapies failed or would not be appropriate.	2 dose levels of crenigacestat (25 mg and 50 mg) were administered orally 3 times weekly (TIW) over a 28-day cycle.	None of the patients received any clinical benefit (n=5).	PMID: 32939607 113
Desmoma	PF-03084014	I	Advanced patients resistant to standard therapy or for which no therapy was available.	The oral dose of PF-03084014 ranges from 20 to 330 mg twice daily.	Of the 7 patients, 5 achieved a PR, with a mean time to achieve a response of 11.9 months. All patients who achieved PR continued responding over 47.9 to 73 months.	PMID: 28887726 121
	PF-03084014	II	Age ≥ 18 years; Patients with histologically confirmed desmoid tumors who were not candidates for surgical resection or definitive radiation therapy and whose disease progressed aggressively after at least one line of standard treatment.	PF-03084014 was taken orally at a dose of 150mg twice daily in 21-day cycles.	Of the 16 patients evaluable, 5 achieved a confirmed partial response and had been on study for more than 2 years, and another 5 patients with prolonged SD remained on study.	PMID: 28350521 122
	LY3039478	I	Age ≥ 20 years; Japanese patients with advanced solid tumors for whom standard therapies failed or would not be appropriate.	2 dose levels of crenigacestat (25 mg and 50 mg) was administered orally 3 times weekly (TIW) over a 28-day cycle.	None of the patients had a complete or partial response to the treatment. One patient with a desmoid tumor in the 50-mg treatment arm showed tumor size shrinkage of 22.4% and had stable disease for 22.5 months.	PMID: 32939607 113
	PF-03084014	I	Advanced patients who were resistant to standard therapy or for which no therapy was available.	Oral administration for 21 days in a test dose range of 20 to 330 mg BID (Please refer to this article for specific usage);	Of the 7 patients evaluable, 5 achieved a partial response (71.4% objective response rate).	PMID: 25231399 106
	Nirogacestat	III	Adults with progressing desmoid tumors	Oral administration in a test dose of 150mg BID	Patients receiving Nirogacestat performed better on progression-free survival, objective response, pain, symptom burden, physical function, role function, and health-related quality of life. Although adverse events with Nirogacestat are frequent, they are mostly low grade.	PMID: 36884323^85^
